# Increased risk of contralateral breast cancer for BRCA1/2 wild-type, high-risk Korean breast cancer patients: a retrospective cohort study

**DOI:** 10.1186/s13058-024-01769-x

**Published:** 2024-01-22

**Authors:** Eunhye Kang, Ji-Jung Jung, Changjin Lim, Hong-Kyu Kim, Han-Byoel Lee, Wonshik Han, Hyeong-Gon Moon

**Affiliations:** 1 Department of Surgery, Seoul National University College of Medicine, 103, Daehak-ro, Jongno-gu, Seoul, 03080 Korea; 2 Genomic Medicine Institute, Seoul National University Medical Research Center, Seoul, Korea; 3 Cancer Research Institute, Seoul National University, Seoul, Republic of Korea

**Keywords:** Breast cancer, Contralateral breast cancer, Overall survival, *BRCA* mutation, *BRCAx*

## Abstract

**Background:**

This study aimed to investigate the contralateral breast cancer (CBC) recurrence rate in Korean breast cancer patients according to their *BRCA1/2* germline mutation status, focusing particularly on the CBC recurrence risk in *BRCA1/2* negative (*BRCAx*) patients.

**Methods:**

We conducted a retrospective study on 13,107 primary breast cancer patients. The patients were divided into high-risk and low-risk groups for hereditary breast cancer based on the Korean National Health Insurance Service’s eligibility criteria for *BRCA1/2* germline mutation testing. The high-risk group was further categorized into the *BRCA* *mutation group*, the *BRCAx* *group*, and the *not tested group*. We evaluated the overall survival and cumulative risk of developing CBC in these patients.

**Results:**

Among 4494 high-risk patients, 973 (21.7%) underwent genetic testing for *BRCA1/2* germline mutation, revealing mutations in 158 patients (16.2%). We observed significant overall survival differences across all four groups, with the high-risk, not-tested group demonstrating notably worse overall survival (*p* < 0.001). However, when adjusted for other prognostic factors, there was no significant differences in hazard ratio of death between the four groups. The cumulative risk of CBC also varied among the groups. Patients with *BRCA1/2* mutations showed a 7.3-fold increased risk of CBC compared to the low-risk group (95% CI 4.11–13.0, *p* < 0.001). Interestingly, *BRCAx* patients also demonstrated a significantly higher risk of CBC (HR 2.77, 95% CI 1.76–4.35, *p* < 0.001). The prognostic importance of the *BRCAx* for CBC recurrence persisted after adjusting for the age and subtype, but became insignificant when the family history of breast cancer was adjusted.

**Conclusion:**

Breast cancer patients who are at high risk of hereditary breast cancer but with wild-type *BRCA 1/2* genes (*BRCAx*) have increased risk of developing contralateral breast cancer when compared to the low-risk patients. More careful surveillance and follow-up can be offered to these patients especially when they have family history of breast cancer.

**Supplementary Information:**

The online version contains supplementary material available at 10.1186/s13058-024-01769-x.

## Background

*BRCA1* and *BRCA2*, the two major genes regulating genome protection at various stages of the DNA damage response and DNA repair, are well-known breast and ovarian cancer-susceptibility genes [1–4]. While the retrospective studies have suggested that the cancer risk might vary between *BRCA1* and *BRCA2* mutation carriers [5], recent prospective studies have shown that the lifetime breast cancer risk is similar for both genes ranging from 55 to 72% [6–8]. For Korean breast cancer patients, the prevalence of *BRCA1/2* mutation for patients with a family history of breast or ovarian cancer is 22.3% [9], and the cumulative risk of breast cancer is 72.1% for *BRCA1* and 66.3% for *BRCA2* mutation carriers [10].

Breast cancer patients with *BRCA1/2* germline mutation carry increased risk of contralateral breast cancer (CBC) development [7, 11]. For Korean patients, a fivefold increase in CBC risk was observed for 132 triple negative breast cancer patients with *BRCA1/2* germline mutation when compared to 868 *BRCA1/2* negative patients [12]. As the incidence of breast cancer for Korean women is constantly rising [13] along the increased use of cancer-susceptibility genetic testing [14], it has become clinically important to assess the individual risk for CBC based on their genetic testing results.

In addition to the *BRCA1/2* germline mutation carriers, recent studies suggest the presence of another clinically distinct group of hereditary breast cancer patients who are *BRCA1/2* negative (*BRCAx*) [15, 16]. While prediction models suggest that the low-penetrance genetic loci which may explain a substantial portion of increased breast cancer risk associated with *BRCAx* [17], there is no data on the oncologic outcomes for Korean *BRCAx* breast cancer patients. In this study, we investigated the rate of CBC recurrence in Korean breast cancer patients according to the *BRCA1/2* germline mutation status. Especially, we determined the relative risk of CBC recurrence in Korean *BRCAx* patients compared to the low-risk breast cancer patients.

## Methods

### Patients

This study was a retrospective study based on the data of the 13,107 patients with primary breast cancer who were treated at Seoul National University Hospital from January 2005 to December 2018 with curative intention. Patients diagnosed with DCIS, male breast cancer, or bilateral breast cancer, as well as those who underwent surgery for palliative purposes or had distant metastasis were excluded. These patients were divided into either the high-risk or low-risk group for hereditary breast cancer by the eligibility criteria for *BRCA1/2* germline mutation testing set by the Korean National Health Insurance Service (KNHIS). KNHIS reimburses the *BRCA1/2* testing when any of the following conditions are met: (1) one or more third-degree relative with breast cancer, ovary cancer, metastatic prostate cancer, and pancreas cancer, (2) age at diagnosis is under 40 years, (3) age at diagnosis is under 60 years with triple negative type breast cancer, (4) diagnosed with ovarian cancer.

The high-risk group was further classified into three groups; *BRCA mutation group, BRCAx group, and not tested group*. Patients in the *BRCA mutation group* were those who had tested for *BRCA 1/2* germline mutation and had a pathogenic or likely pathogenic gene mutation. Patients in the *BRCAx group* were those who had a high risk of hereditary breast cancer but had tested negative for *BRCA 1/2* mutation or had a variant of uncertain significance (VUS) mutation. Finally, the patients in the *not tested group* were those who had not tested for *BRCA1/2* mutation in high-risk group. The criteria for classifying high-risk groups into *BRCA mutation group, BRCAx group, and not tested group* were based on the test results performed prior to the occurrence of contralateral breast cancer. Patients who underwent *BRCA* testing after the occurrence of contralateral breast cancer were classified into the *not tested group* regardless of the test results.

We reviewed the clinical and pathologic characteristics, family history information, and the oncologic outcomes of the study subjects. We used following definitions for family history. *Family history* was defined as third-degree relative with breast cancer, ovary cancer, metastatic prostate cancer, and pancreas cancer, *family history of breast cancer* was defined as third-degree relative with only breast cancer, and *first-degree relative of breast cancer* was defined as parents, siblings, or children who were diagnosed with breast cancer.

Among all patients, 973 patients underwent *BRCA* testing. One patient with *BRCA1* germline mutation underwent *BRCA* testing prior to the initial diagnosis due to her family history of breast cancer. The remaining 972 patients underwent blood sampling after their diagnosis of breast cancer with the median time from diagnosis to blood draw being 2.1 months (range 0–168 months).

This study was approved by the Institutional Review Board (IRB) of Seoul National University Hospital (IRB No. 2208-056-1349).

### Statistical analysis

For intergroup comparisons, t test and ANOVA test were used for continuous variables, and Chi-square was using for descriptive data. Cumulative risk of contralateral breast cancer was assessed by Kaplan–Meier curves and log rank tests in each group. The Cox proportional hazard model was used for calculating hazard ratios. The beginning of follow-up was set as the date of breast surgery. Follow-up time of patients without an event of interest was censored at the date of their last contact. In this study, only metachronous contralateral breast cancers diagnosed at least 3 months after the initial breast surgery were defined as contralateral breast cancer events. Both ductal carcinoma in situ and invasive contralateral cancers were included. An overall survival event was defined as death due to any cause. For patients experiencing either a contralateral breast cancer event or overall survival event, the end of follow-up was defined as the date of the event. A *p* value < 0.05 was considered statistically significant. Statistical analyses were conducted using SPSS 25.0 software and R version 4.1.2.

## Results

### Demographics and clinicopathologic characteristics

**Fig. 1:**
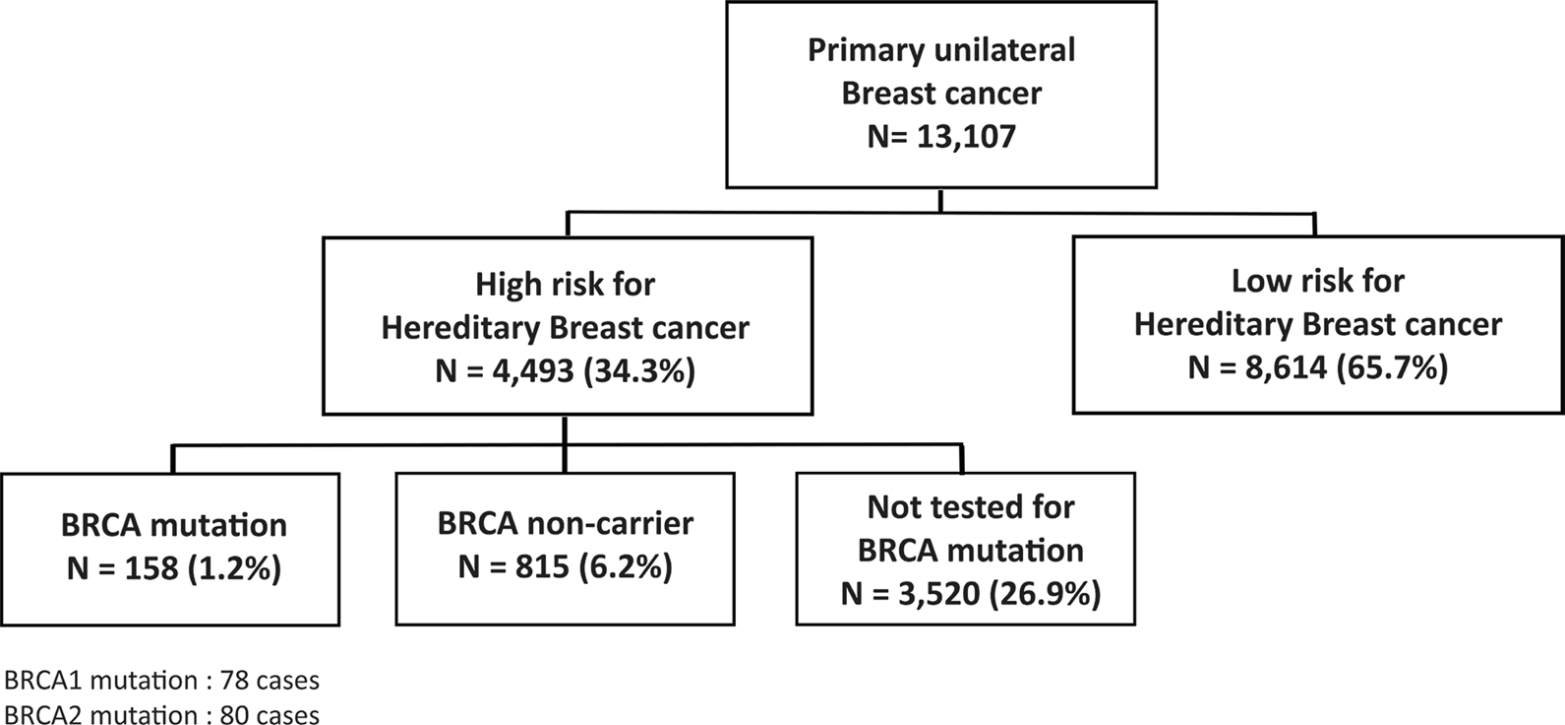
Baseline demographics

The number of patients in each group is shown in Fig. 1. Among the 13,107 patients who met the inclusion criteria, 4493 (34.3%) and 8614 (65.7%) patients were classified as high- and low-risk of carrying *BRCA1/2* germline mutations, respectively. The clinicopathologic characteristics of high- and low-risk patients are shown in Table 1. Notably, the high-risk patients were often associated with unfavorable features including younger age at diagnosis, advanced tumor stages, high histologic grade, and hormone receptor negativity.

**Table 1:** Clinicopathologic characteristics of all patients

	Low risk (*N* = 8614)	High risk (*N* = 4493)	*p* value
Age	52.0 [47.0;60.0]	41.0 [37.0;51.0]	< 0.001
Location			0.306
Right	4212 (48.9%)	2240 (49.9%)	
Left	4402 (51.1%)	2253 (50.1%)	
Breast surgery			< 0.001
Breast conserving surgery	5297 (61.5%)	3002 (66.8%)	
Mastectomy	3317 (38.5%)	1491 (33.2%)	
Axilla surgery			< 0.001
Sentinel LN biopsy	5255 (61.0%)	2576 (57.3%)	
Axilla LN dissection	3111 (36.1%)	1820 (40.5%)	
Not done	49 (0.6%)	12 (0.3%)	
Unknown	199 (2.3%)	85 (1.9%)	
T stage			< 0.001
T1	4477 (52.0%)	1898 (42.2%)	
T2	3447 (40.0%)	2138 (47.6%)	
T3	485 (5.6%)	321 (7.1%)	
T4	198 (2.3%)	126 (2.8%)	
N stage			< 0.001
N0	5100 (59.2%)	2478 (55.2%)	
N1	2245 (26.1%)	1236 (27.5%)	
N2	834 (9.7%)	511 (11.4%)	
N3	391 (4.5%)	247 (5.5%)	
Subtype			< 0.001
Hormone receptor+/HER2−	6181 (71.8%)	2020 (45.0%)	
Hormone receptor+/HER2+	965 (11.2%)	406 (9.0%)	
Hormone receptor−/HER2+	1114 (12.9%)	263 (5.9%)	
Hormone receptor−/HER2−	354 (4.1%)	1804 (40.2%)	
Histologic grade			< 0.001
1	936 (10.9%)	253 (5.6%)	
2	4477 (52.0%)	1712 (38.1%)	
3	2745 (31.9%)	2239 (49.8%)	
Unknown	456 (5.3%)	289 (6.4%)	
Lymphovascular invasion			< 0.001
Present	2388 (27.7%)	1409 (31.4%)	
None	5933 (68.9%)	2886 (64.2%)	
Unknown	293 (3.4%)	198 (4.4%)	

Among the 4493 high-risk patients, 973 (21.7%) patients underwent genetic testing for germline *BRCA1/2* mutation. Genetic testing revealed *BRCA1/2* germline mutation in 158 (16.2%) patients. The remaining 815 patients (83.8%), who were determined to be high risk but genetic testing showed *BRCA1/2* wild type, comprised the *BRCAx* group. The rates for *BRCA1/2* genetic testing varied by the clinical indications (*p* < 0.001). Patients with a family history of breast cancer or personal history of ovarian cancer had higher rates of germline *BRCA1/2* testing (41.4% and 57.9%, respectively, Additional file 1: Table S1).

The clinicopathologic characteristics of the high-risk group patients are shown in Table 2. Compared to the *BRCAx* group or high-risk not-tested group, patients with *BRCA* mutations had significantly higher incidences of ovarian cancer and family history of breast cancer (*p* < 0.001). While the three groups showed no significant difference in tumor size, nodal status, or histologic grade, the distribution of molecular subtypes showed statistically significant differences.

**Table 2:** Clinicopathologic characteristics of high-risk patients

	*BRCA mutation* (*N* = 158)	*BRCAx* (*N* = 815)	*Not tested* (*N* = 3520)	*p* value
Median age [IQR]	40.5 [35.0;51.0]	39.0 [35.0;50.0]	41.0 [37.0;51.0]	< 0.001
Ovary cancer				< 0.001
Yes	7 (4.4%)	15 (1.8%)	16 (0.5%)	
No	151 (95.6%)	800 (98.2%)	3504 (99.5%)	
Family history				< 0.001
Yes	116 (73.4%)	421 (51.7%)	761 (21.6%)	
No	40 (25.3%)	386 (47.4%)	2631 (74.7%)	
Unknown	2 (1.3%)	8 (1.0%)	128 (3.6%)	
Family history of breast cancer				< 0.001
Yes	103 (65.2%)	403 (49.4%)	667 (18.9%)	
No	53 (33.5%)	404 (49.6%)	2725 (77.4%)	
Unknown	2 (1.3%)	8 (1.0%)	128 (3.6%)	
First-degree relative with breast cancer				< 0.001
Yes	79 (50.0%)	336 (41.2%)	457 (13.0%)	
No	76 (48.1%)	471 (57.8%)	2931 (83.3%)	
Unknown	3 (1.9%)	8 (1.0%)	132 (3.8%)	
T stage				0.527
T1	60 (38.0%)	366 (44.9%)	1472 (41.8%)	
T2	83 (52.5%)	376 (46.1%)	1679 (47.7%)	
T3	9 (5.7%)	51 (6.3%)	261 (7.4%)	
T4	5 (3.2%)	21 (2.6%)	101 (2.9%)	
Unknown	1 (0.6%)	1 (0.1%)	7 (0.2%)	
N stage				0.603
N0	78 (49.4%)	445 (54.6%)	1955 (55.5%)	
N1	47 (29.7%)	226 (27.7%)	963 (27.4%)	
N2	19 (12.0%)	90 (11.0%)	402 (11.4%)	
N3	13 (8.2%)	52 (6.4%)	183 (5.2%)	
Unknown	1 (0.6%)	2 (0.2%)	17 (0.5%)	
Histologic grade				< 0.001
1	4 (2.5%)	69 (8.5%)	180 (5.1%)	
2	50 (31.6%)	402 (49.3%)	1260 (35.8%)	
3	97 (61.4%)	308 (37.8%)	1834 (52.1%)	
9	7 (4.4%)	36 (4.4%)	246 (7.0%)	
Lymphovascular invasion				0.671
Present	53 (33.5%)	248 (30.4%)	1108 (31.5%)	
None	99 (62.7%)	537 (65.9%)	2250 (63.9%)	
Unknown	6 (3.8%)	30 (3.7%)	162 (4.6%)	
Subtype				< 0.001
HR+/HER2−	75 (47.5%)	488 (59.9%)	1457 (41.4%)	
HR+/HER2+	6 (3.8%)	112 (13.7%)	288 (8.2%)	
HR−/HER2+	6 (3.8%)	53 (6.5%)	204 (5.8%)	
HR−/HER2−	71 (44.9%)	162 (19.9%)	1571 (44.6%)	

*IQR* interquartile range, *HR* hormone receptor

### Overall survival and the cumulative risk of CBC

**Fig. 2:**
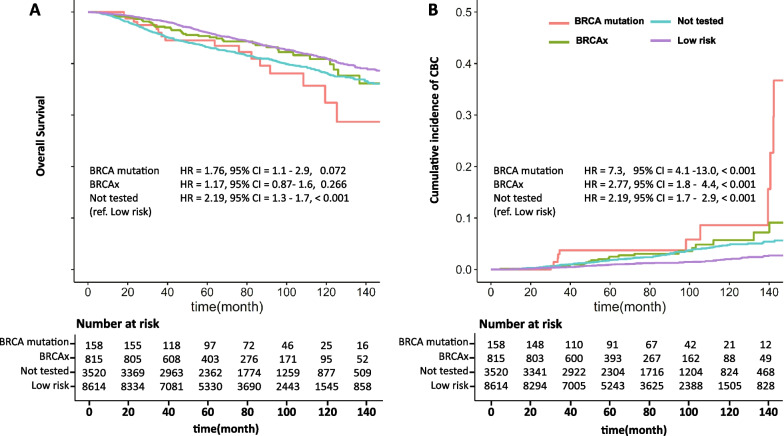
Cumulative risk of contralateral breast cancer and overall survival in each group

As shown in Fig. 2A, the four groups of patients showed significant overall survival differences. (The 5-year and 10-year overall survival for each group is presented in Additional file 1: Table S2.) The median duration of follow-up for the patients was 72.6 months. When compared to the low-risk group, high-risk *not tested group* showed significantly worse overall survival outcome (*p* < 0.001). The *BRCA1/2* *mutation group* showed worse overall survival compared to the low-risk group (HR 1.17, 95% CI: 1.1–2.9); however, this difference was not statistically significant (*p* = 0.072). However, when adjusted for other prognostic factors, there was no significant differences in hazard ratio of death between the four groups (Table 3).

**Table 3:** Hazard ratio of mortality from univariate and multivariate cox regression model

Variable	Univariate			Multivariate		
	HR	[95% CI]	*p* value	HR	[95% CI]	*p* value
Age						
Age > 40	1					
Age ≤ 40	1.33	[1.14, 1.55]	< 0.001			
TNBC						
Yes	2.43	[2.11, 2.79]	< 0.001	1.44	[1.16, 1.79]	< 0.001
Ovary cancer						
Yes						
First-degree relatives with BC						
Yes	0.7	[0.51, 0.95]	0.023	0.84	[0.59, 1.18]	0.315
T stage	2.24	[2.09, 2.41]	< 0.001	1.56	[1.25, 1.95]	< 0.001
N stage	2.06	[1.93, 2.19]	< 0.001	1.71	[1.47, 1.75]	< 0.001
Histologic grade	1.08	[1.05, 1.11]	< 0.001			
Endocrine therapy						
Yes	0.37	[0.33, 0.42]	< 0.001	0.53	[0.44, 0.63]	< 0.001
*BRCA* test						
Low risk	1			1		
*Not tested*	1.46	[1.27, 1.68]	< 0.001	0.88	[0.74, 1.05]	0.165
* BRCAx*	1.18	[0.87, 1.58]	0.282	0.92	[0.66, 1.27]	0.611
* BRCA* *mutation*	1.76	[1.07, 2.89]	0.026	1.02	[0.59, 1.77]	0.944

The cumulative risk of CBC also varied among the four groups (Fig. 2B). As expected, the patients with germline *BRCA1/2* mutation showed 7.3-fold increase of CBC risk when compared to the low-risk group (*p* < 0.001). Also, the high-risk *not tested group* showed significant increase in CBC risk (*p* < 0.001). Interestingly, the patients in the *BRCAx* *group* who had wild-type *BRCA1/2* also showed significantly higher risk of CBC when compared to the low-risk group with the hazard ratio of 2.77 (*p* < 0.001). The prognostic importance of the *BRCAx* for CBC recurrence persisted after adjusting for the age and subtype, but became insignificant when the family history of breast cancer was adjusted (Table 4).

**Table 4:** Hazard ratio of contralateral breast cancer derived from univariate and multivariate cox regression model

Variable	Univariate			Multivariate (including family history)			Multivariate (excluding family history)		
	HR	[95% CI]	*p* value	HR	[95% CI]	*p* value	HR	[95% CI]	*p* value
Age									
Age > 40	1			1			1		
Age ≤ 40	1.68	[1.27, 2.22]	< 0.001	1.25	[0.85, 1.84]	0.26	0.98	[0.85, 1.84]	0.26
TNBC									
Yes	2.18	[1.66, 2.87]	< 0.001	1.69	[1.20, 2.40]	0.003	1.44	[1.02, 2.02]	0.036
Ovary cancer									
Yes	1.06	[0.15, 7.55]	0.955						
First-degree relatives with BC									
Yes	2.92	[2.08, 4.08]	< 0.001	1.95	[1.23. 3.08]	0.004			
T stage	0.95	[0.79, 1.14]	0.575						
N stage	1.02	[0.88, 1.19]	0.796						
Histologic grade	1.16	[0.93, 1.44]	0.182						
Endocrine therapy									
Yes	0.48	[0.37, 0.61]	< 0.001						
*BRCA* test									
Low risk	1			1			1		
Not tested	2.19	[1.67, 2.86]	< 0.001	1.41	[0.92, 2.16]	0.115	1.88	[1.29, 2.76]	0.001
* BRCAx*	2.77	[1.76, 4.35]	< 0.001	1.57	[0.84, 2.91]	0.154	2.6	[1.56, 1.33]	< 0.001
* BRCA* mutation	7.3	[4.11, 13.0]	< 0.001	3.2	[1.44, 7.14]	0.004	6.17	[3.23, 11.8]	< 0.001

*TNBC* triple negative breast cancer, *HR* hazard ratio, *CI* confidence interval, *BC* breast cancer

## Discussion

The present study demonstrates that, in addition to the breast cancer patients with germline *BRCA1/2* mutation, the patients with wild-type *BRCA1/2* who are high-risk of having hereditary breast cancer (*BRCAx)* also carry an increased risk of CBC recurrence when compared to that of low-risk patients. The increased risk of CBC in high-risk breast cancer patients with wild-type *BRCA1/2* seems mostly due to having the family history.

Previous studies have examined differences in CBC risk between patients with *BRCA* mutations and non-carriers within high-risk cohorts [18, 19] or between sporadic patients and *BRCA* mutation carriers [20, 21]. In contrast, our study directly compared the CBC risk among patients with high risk for hereditary breast cancer, sporadic patients, *BRCA* mutation carriers, and *BRCAx* group within a relatively large cohort treated at a single institution. Our findings are meaningful because they demonstrate that patients with high-risk factors, even in the absence of *BRCA* mutations, have a higher CBC cumulative risk compared to low-risk sporadic patients.

There are several studies that have investigated the cumulative risk of contralateral breast cancer in patients with confirmed *BRCA* non-carriers (*BRCAx* group), but the results are inconsistent, and have shown varying results. There are studies that suggest that non-carriers of *BRCA* mutations have a higher risk of developing contralateral breast cancer compared to sporadic patients. Reiner et al*.* showed that *BRCA* non-carriers with family history breast cancer were at significantly greater risk of CBC than other breast cancer survivors. The 10-year cumulative risks of developing breast cancer for those without a family history, with only second-degree family history, and with first-degree family history were 4.6%, 5.9%, and 8.6%, respectively. Moreover, non-carriers with a bilaterally affected first-degree relative have a 10-year cumulative risk of CBC that is nearly as high as that of *BRCA* mutation carriers (15.6% vs. 18.4%, respectively) [22]. In other study, Yoon et al. showed that non-carriers with high risk of hereditary breast cancer patients have also been found to have a higher risk of CBC, the 10-year cumulative risk for CBC was 9.8% for non-carriers, 23.8% for *BRCA1* mutation carriers, and 19.1% for *BRCA2*. There was no statistically significant difference in CBC risk between *BRCA* mutation carriers and non-carriers [19].

However, several studies have shown that *BRCAx* patients do not have significantly different CBC risks compared to sporadic (without family history of breast cancer) breast cancer patients [23, 24]. Tilanus-Linthorst et al*.* argued that the reports of higher CBC incidence and better survival in non*-BRCA1/2* patients may be substantially influenced by selection bias due to DNA testing. Patients who already had contralateral breast cancer or were at higher risk of developing CBC were more likely to undergo BRCA gene testing, which could have influenced the results [25].

One possible explanation for the high incidence of contralateral breast cancer (CBC) in the *BRCAx* group is that there may be mutations in high-penetrance genes other than *BRCA1/2*, such as *PTEN*, *CDH1*, and *CHEK2*, or the presence of common low-penetrance variants that increase the risk of developing cancer in the contralateral breast. A study of Korean *BRCAx* patients found that 4.2% of the overall patients were affected by moderate-/high-penetrance variants, and showed that high-risk breast cancers, particularly for Asians, might consist of multiple layers with similar importance, moderate/high-penetration genes, and selected common variants [17]. However, Reiner et al. showed that family history of breast cancer remains a strong risk factor for CBC, even after excluding carriers of deleterious mutations in *BRCA1, BRCA2, ATM, CHEK2 or PALB2*, and after adjusting for 67 common breast cancer-susceptibility single nucleotide polymorphisms (SNPs) [26]. This suggests that there may be other factors at play beyond genetic ones. A second possible explanation is that patients with a familial history of breast cancer may be influenced by environmental factors that contribute to the development of breast cancer, in addition to genetic factors. A study by Couto et al*.* estimated that the heritable component of familial breast cancer was 73%, with the environmental proportion at 27% [27]. A third possible explanation is that a large proportion of the *BRCAx* group consists of young patients who have a higher risk of developing breast cancer, which may also increase their risk of developing CBC. Prospective studies have shown that only 5–12% of all women younger than 40 years with a first breast cancer diagnosis were carriers of the *BRCA1 or BRCA2* mutation [28, 29]. Apart from genetic factors, many young breast cancer patients have multiple risk factors associated with breast cancer., such as lean body mass, reproductive factors, and therapeutic radiation, which may also increase their risk of developing CBC for the same reasons [30].

In our study, patients with *BRCA1* mutation and *BRCA2* mutation had a 10-year cumulative CBC risk of 9.85% and 7.20%, respectively, which is lower compared to the results of previous studies (Additional file 1: Table S3). Studies of populations in the USA or Europe have shown that *BRCA1* and *BRCA2* mutations have a 24–35% and 19–29% risk of developing contralateral breast cancer within 10 years of their first breast cancer [8, 31–33]. Research involving patients in Asia has shown a lower cumulative risk of CBC compared to studies on Western patients, yet the risk is higher than that found in our study. Also, the range of risk was broader in these studies, with a 10-year cumulative risk ranging from 15.5 to 26%. The reason the cumulative risk appears lower in our study is that only around 20% of high-risk patients underwent *BRCA* gene testing and the acceptance rate for triple negative breast was lower. This may lead to an underestimation of the actual risk.

Our study has several limitations. First, we did not perform *BRCA* testing on unselected breast cancer patients, which may introduce selection bias in the patients who underwent testing. Second, although we had a relatively large number of patients with *BRCA* mutations compared to previous Asian studies, the number is still smaller and the follow-up period is shorter than in Western studies. Therefore, further analyses with a larger number of patients and long-term follow-up are needed in the future.

## Conclusion

Breast cancer patients who are at high-risk of hereditary breast cancer but with wild-type *BRCA 1/2* genes (*BRCAx*) have increased risk of developing contralateral breast cancer when compared to the low-risk patients. More careful surveillance and follow-up can be offered to these patients especially when they have family history of breast cancer.

### Supplementary Information

**Additional file 1: Table S1.** The acceptance rate of BRCA tests conducted based on different testing criteria. **Table S2.** Description: 5-year and 10-year overall survival in each group. **Table S3.** 5-year and 10-year cumulative incidence of contralateral breast cancer.

## Data Availability

The datasets used and/or analyzed during the current study are available from the corresponding author on reasonable request.

## Abbreviations

CBC: Contralateral breast cancer
LN: Lymph node
HR: Hormone receptor
TNBC: Triple negative breast cancer
HR: Hazard ratio
CI: Confidence interval
BC: Breast cancer
