# Supplementary material for: Increased risk of contralateral breast cancer for BRCA1/2 wild-type, high-risk Korean breast cancer patients: a retrospective cohort study
Source: Breast Cancer Res. 2024 Jan 22;26:14. doi: 10.1186/s13058-024-01769-x (PMC10801954; doi:10.1186/s13058-024-01769-x)
Supplement: Supplementary file 1 — Additional file 1: Table S1. The acceptance rate of BRCA tests conducted based on different testing criteria. Table S2. Description: 5-year and 10-year overall survival in each group. Table S3. 5-year and 10-year cumulative incidence of contralateral breast cancer. [file 13058_2024_1769_MOESM1_ESM.docx]

**Additional file 1**

Table S1. Acceptance rate of the *BRCA1/2* testing.

|  | **No. of meet criteria** | **No. of BRCA testing** | **rate** |
| --- | --- | --- | --- |
| Total | 4,493 | 973 | 21.7% |
| Family history (3^rd^ degree) | 1,298 | 537 | 41.4% |
| 1st degree relative with breast cancer | 872 | 415 | 47.6% |
| Young age (≤40) | 2,219 | 518 | 23.3% |
| TNBC & ≤ 60 years old | 1,760 | 219 | 12.4% |
| Ovary cancer | 38 | 22 | 57.9% |

TNBC: triple negative breast cancer

Table S2. 5-year and 10-year overall survival in each group.

|  | ***BRCA1* mutation** | ***BRCA2* mutation** | ***BRCAx*** | **not tested** | **low risk** | **total** |
| --- | --- | --- | --- | --- | --- | --- |
| 5 year | 95.2% | 97.2% | 97.5% | 98.2% | 99.1% | 98.7% |
| 10 year | 80.2% | 85.3% | 90.9% | 88.2% | 91.0% | 90.1% |

Table S3. 5-year and 10-year cumulative incidence of contralateral breast cancer.

|  | ***BRCA1* mutation** | ***BRCA2* mutation** | ***BRCAx*** | **not tested** | **low risk** | **total** |
| --- | --- | --- | --- | --- | --- | --- |
| 5 year | 4.84% | 2.80% | 2.50% | 1.80% | 0.90% | 1.3% |
| 10 year | 9.85% | 7.20% | 5.69% | 4.80% | 2.00% | 3.1% |
